# Editorial: Oxidative Stress in Cardiovascular Diseases and Pulmonary Hypertension

**DOI:** 10.3389/fcvm.2022.868988

**Published:** 2022-03-23

**Authors:** Nirmal Parajuli, Djuro Kosanovic

**Affiliations:** ^1^Immunology Research Program, Henry Ford Health System, Detroit, MI, United States; ^2^Department of Pulmonology, I. M. Sechenov First Moscow State Medical University (Sechenov University), Moscow, Russia

**Keywords:** oxidative stress, pulmonary disease, cardiovascular disease, chronic obstructive pulmonary disease, pulmonary hypertension, reactive oxygen species

Cardiovascular and pulmonary diseases are the leading causes of death worldwide (1, 2). Various pathogenic risk factors, including oxidative stress have detrimental effects on the heart and lung tissues, thereafter, causing changes to various disease states (3, 4). In general, oxidative stress leads to an imbalance between the production of reactive oxygen species (ROS) and antioxidant defenses. The production of ROS is dependent on various enzymes, including the nicotinamide adenine dinucleotide phosphate (NADPH) oxidase (NOXs), xanthine oxidase, and the mitochondrial electron transport chain (5). Under physiological conditions, the concentrations of ROS are subtlety regulated by numerous antioxidant enzymes such as catalase, peroxiredoxins, thioredoxins, glutaredoxins, glutathione peroxidases, and glutathione (6, 7). Apart from ROS, another free radical of reactive nitrogen species, e.g., peroxynitrite, forms from the binding of nitric oxide (NO) to superoxide and plays a complex role in the pathophysiology of various cardiovascular and pulmonary diseases (8, 9). The NO production involves a nitric oxide synthase (NOS) enzyme that consists of two constitutive neuronal and endothelial NOS as well as another inducible one. Generally, the reactive peroxynitrite uncouples eNOS and switches to superoxide production from NO (10). The physiological interplay of NO or superoxide is indispensable for modulating vascular tone, airway tone, metabolism, and immune responses. Despite this importance, any derailment of NO or superoxide generation may play a pivotal role in the development and establishment of numerous cardiac and pulmonary disease conditions (4, 11). Based on the importance of oxidative stress, our contributors provided their foremost current scientific update by review and research articles describing molecular crosstalk as well as preclinical findings.

Over the years, various studies suggest that a sedentary lifestyle increases vascular NOXs and enhances vascular ROS production (12–14). Additionally, this lifestyle accumulates visceral fat that activates several inflammatory pathways (15). Conversely, regular physical exercise may counteract metabolic disorder and provides cardiorespiratory benefit by lowering oxidative stress (16, 17). To examine this, some studies identified that physical exercise enhances the activities of numerous transcription factors (TF) including nuclear factor kappa B (NF-κB), activator protein 1 and peroxisome proliferator-activated receptor-gamma coactivator-1α. These activities provide antioxidant defense by upregulating antioxidant enzymes and decreasing NOXs functions for eventual reduction of oxidative stress (7). Despite the advantage of exercise, prolonged intense aerobic training can result in decreased NO bioavailability, which may impair endothelial-dependent vasodilation through decrease antioxidants and increase ROS, thereby mediating cardiovascular abnormalities (18–20). Given the aforementioned role of NO in exercise, Parshukova et al. analyzed the NO production in professional cross-country skiers during physical activity at maximum load and their results suggest that decreased NO in plasma may become a potential avenue to diagnose endothelial dysfunction for professional athletes. It is well-established that decreased endothelial NO production may link to increased oxidative stress, endothelial dysfunction in pulmonary vascular bed, pulmonary vascular remodeling, and loss of precapillary vessels (10, 21, 22). Moreover, these changes occur with the infiltration of macrophages that lead to the production of numerous inflammatory markers for aggravating pulmonary artery remodeling and pulmonary arterial hypertension (PAH) (23). In addition, sympathetic and parasympathetic abnormalities have been well-documented in PAH (14, 15). The enhanced activation of parasympathetic and reduced sympathetic activities can help against PAH progression (14, 15). The activation of parasympathetic nervous system may exert protective effects in cardiopulmonary diseases through cholinergic anti-inflammatory pathway by controlling innate immune responses (24). To better understand the involvement of the parasympathetic in pulmonary diseases, Qiu et al. demonstrated that donepezil, an oral cholinesterase inhibitor therapy, attenuates pulmonary vascular and right ventricular remodeling by enhancing parasympathetic activity in a monocrotaline-induced PAH rat model. Additionally, these authors demonstrated that donepezil mediated effects for reducing hyper-proliferation and apoptosis-resistant phenotype of pulmonary arterial smooth muscle cells in PAH by suppressing the activation of M2-macrophage immune cells.

Immune cells, including macrophages, express both adrenergic and nicotinic receptors that bind with neurotransmitters such as acetylcholine released by sympathetic and parasympathetic nerve endings in order to initiate immune-modulatory responses (25). Both the sympathetic and parasympathetic nervous systems are influential in producing neuro-immune processes (25). It is well-established that macrophages are effector cells during an innate immune response. The innate immune response calls the adaptive immune response into play. Both immune systems work together to make antibodies that act independently against extracellular pathogens and toxins (26). Although sometimes an overactive immune system can generate antibodies that are specific to self-molecules or tissues which are referred to as autoantibodies (27). Formation of these autoantibodies has now been recognized as a key factor for the high prevalence of PAH patients (28, 29). In line with previously published data, Shu et al. summarized the importance of the lung based cell specific immune response, the potential auto-antigens and the modulating role of local immunoglobulin in pathogenesis of PAH including the development of precise therapy in PAH patients. Most intriguingly, recent studies have indicated that patients with common lung diseases, including chronic obstructive pulmonary disease (COPD) are more likely to develop pulmonary hypertension (PH) and other cardiovascular diseases (30, 31). To increase the understanding about COPD, Karnati et al. summarized the role of oxidative stress in COPD and PH by describing a detailed description on the pathogenesis of pulmonary vascular remodeling. Additionally, authors have highlighted the oxidative/nitrosative stress mediated abnormalities in pulmonary vascular bed and its relationship to the inflammation, endothelial dysfunction, dysregulated proliferation/apoptosis as well as the potential therapeutic measure.

Next, published evidence indicates that COPD patients are at increased risk of suffering from various cardiovascular diseases including heart failure, ischemic heart, and hypertension (31, 32). Interestingly, about one third of patients with COPD are obese (32, 33). To better understand the role of obesity in vascular dysfunction, Zhou et al. summarized the current understanding of the relationship between oxidative stress in obesity and vascular endothelial dysfunction. In this review, they described the possible risk factors of oxidative stress in obesity, and the impact of obesity-induced oxidative stress on adipose tissue function. Additionally, their review highlights the crosstalk between adipose tissue and vasculature mediated by adipocytokines as well as the potential target mediating adipose tissue oxidative stress.

With the significant increase of understanding about obesity in scientific research, adipose tissue is now considered as a central metabolic organ in the regulation of whole-body energy homeostasis by lipid metabolism (34, 35). It has been well-characterized that excessive alcohol consumption, also known as binge drinking results in dysregulated lipid metabolism within adipose tissue (36). To better understand this process, Seidel et al. focussed their research work on the role of binge drinking on specific S-glutathionylation in the aorta, liver, and brain by using an ApoE deficient mouse model. Their findings reported that binge drinking led to aorta- and liver-specific regulation of the glutathionylation regulatory enzyme system, eliciting decreased glutaredoxin-1 and increased glutathione S-transferase. Precisely, they suggested that the activation of aorta- and liver-based S-glutathionylation compromises aortic endothelial dysfunction and fatty liver, which might be a potential underlying mechanism of increased risk factor for cardiovascular diseases among binge drinkers. Understanding the complexity of oxidative stress, Wang et al. summarized the mechanistic role of ROS on various intracellular signaling such as toll like receptor-4, nuclear factor kappa B cells, mitogen-activated protein kinase, CD26, heme oxygenase-1, transient receptor potential ion channels and L-type voltage-gated calcium channel in numerous diseases such as diabetes mellitus, hypertension, and ischemia-reperfusion injury.

In conclusion, the above-cited articles for this Research Topic indicate the current ideas and perspectives on the clinical impact from bench side research on the role of oxidative stress that plays in cardiovascular and pulmonary diseases. We believe that these articles provide a significant contribution of new ideas and advancements in the medical fields. We are grateful to our all contributors for sharing their important work for this Research Topic.

## Author Contributions

Both authors wrote, drafted, read, and approved the manuscript.

## Conflict of Interest

The authors declare that the research was conducted in the absence of any commercial or financial relationships that could be construed as a potential conflict of interest.

## Publisher's Note

All claims expressed in this article are solely those of the authors and do not necessarily represent those of their affiliated organizations, or those of the publisher, the editors and the reviewers. Any product that may be evaluated in this article, or claim that may be made by its manufacturer, is not guaranteed or endorsed by the publisher.
